# MUC1-C nuclear localization drives invasiveness of renal cancer cells through a sheddase/gamma secretase dependent pathway

**DOI:** 10.18632/oncotarget.1768

**Published:** 2014-02-02

**Authors:** Audrey Bouillez, Viviane Gnemmi, Kelly Gaudelot, Brigitte Hémon, Bélinda Ringot, Nicolas Pottier, François Glowacki, Caroline Butruille, Christelle Cauffiez, Malika Hamdane, Nicolas Sergeant, Isabelle Van Seuningen, Xavier Leroy, Sébastien Aubert, Michaël Perrais

**Affiliations:** ^1^ Inserm, UMR837, Equipe 5 “Mucines, différenciation et cancérogenèse épithéliales”, Jean-Pierre Aubert Research Center, Lille Cedex, France; ^2^ Faculté de Médecine, Université de Lille 2, Lille Cedex, France; ^3^ Institute of Pathology, Centre de Biologie-Pathologie, CHRU de Lille, Lille Cedex, France; ^4^ EA4483, Faculté de Médecine, Pole Recherche, Lille Cedex, France; ^5^ Department of Nephrology, CHRU de Lille, Lille Cedex, France; ^6^ Department of Urology, CHRU de Lille, Lille Cedex, France; ^7^ Inserm, UMR837, Equipe 1 “Alzheimer and Tauopathies”, Jean-Pierre Aubert Research Center, Lille Cedex, France

**Keywords:** MUC1, kidney cancer, ADAM10, ADAM17, γ-secretase, invasion

## Abstract

MUC1 is a membrane-anchored mucin and its cytoplasmic tail (CT) can interact with many signaling pathways and act as a co-transcription factor to activate genes involved in tumor progression and metastasis. MUC1 is overexpressed in renal cell carcinoma with correlation to prognosis and has been implicated in the hypoxic pathway, the main renal carcinogenetic pathway. In this context, we assessed the effects of MUC1 overexpression on renal cancer cells properties. Using shRNA strategy and/or different MUC1 constructs, we found that MUC1-extracellular domain and MUC1-CT are involved in increase of migration, cell viability, resistance to anoikis and in decrease of cell aggregation in cancer cells. Invasiveness depends only on MUC1-CT. Then, by using siRNA strategy and/or pharmacological inhibitors or peptides, we showed that sheddases ADAM10, ADAM17 and gamma-secretase are necessary for MUC1 C-terminal subunit (MUC1-C) nuclear location and in increase of invasion property. Finally, MUC1 overexpression increases ADAM10/17 protein expression suggesting a positive regulatory loop. In conclusion, we report that MUC1 acts in renal cancer progression and MUC1-C nuclear localization drives invasiveness of cancer cells through a sheddase/gamma secretase dependent pathway. MUC1 appears as a therapeutic target by blocking MUC1 cleavage or nuclear translocation by using pharmacological approach and peptide strategies.

## INTRODUCTION

MUC1 is a large *O*-glycoprotein type I translated as a single polypeptide that undergoes autocleavage into N-terminal (MUC1-N) and C-terminal (MUC1-C) subunits allowing the formation of a heterodimer through a stable non-covalent association [1]. In adult, MUC1 expression is cell- and tissue-specific and is altered during carcinogenesis. The MUC1-N is an extracellular domain containing extensively *O*-glycosylated tandem repeat 20 amino acid (AA) sequence and protudes far away from the apical side of the cell (200–500 nm). The MUC1-C includes a 58-AA extracellular domain, a 28-AA transmembrane domain and a 72-AA cytoplasmic tail (CT) [1]. MUC1-N may be released from cell-surface by a mechanism dependent on at least two sheddases, TACE/ADAM17 [2] and MT1-MMP/MMP14 [3]. MUC1-C (i) is also a substrate for γ-secretase [4] and several kinases such as Src, GSK3β, PI3K [1], (ii) can interact with tyrosine kinase receptors such as EGFR and (iii) plays a role in signal transduction. Nuclear transport of MUC1-CT is dependent on the CQC motif which is required for its dimerization and direct interaction with importin β and nucleoporin 62 [1]. MUC1-CT acts as a co-transcription factor to activate genes involved in tumor progression and metastasis [1].

Renal cell carcinoma corresponds to 5% of all adult malignancies and originates from renal tubules. The main histologic subtype is represented by clear renal cell carcinoma (cRCC; [5]). Ninety percent of cRCC present a biallelic inactivation of the von Hippel Lindau (VHL) tumor suppressor gene resulting in constitutive activation of hypoxia signaling pathway *via* the Hypoxia Inducible Factor (HIF)−1 transcription factor that contributes to the physiology of tumours [6, 7]. cRCC is typically highly resistant to conventional systemic therapies. Previous studies have shown that MUC1 is diffusely overexpressed in cRCC [8, 9] and MUC1 overexpression has been found to be associated with metastatic disease and a worse prognosis [10, 11]. MUC1 is a target gene of HIF-1 [11] but also a regulator of its activity [12, 13].

The purpose of this article was to better understand (a) the roles of MUC1 overexpression on renal cancer cells properties *in vitro* and *in vivo* and (b) the mechanism involved in MUC1-C nuclear localization.

## RESULTS

### Roles of MUC1 in renal cancer cell properties

**Figure 1:**
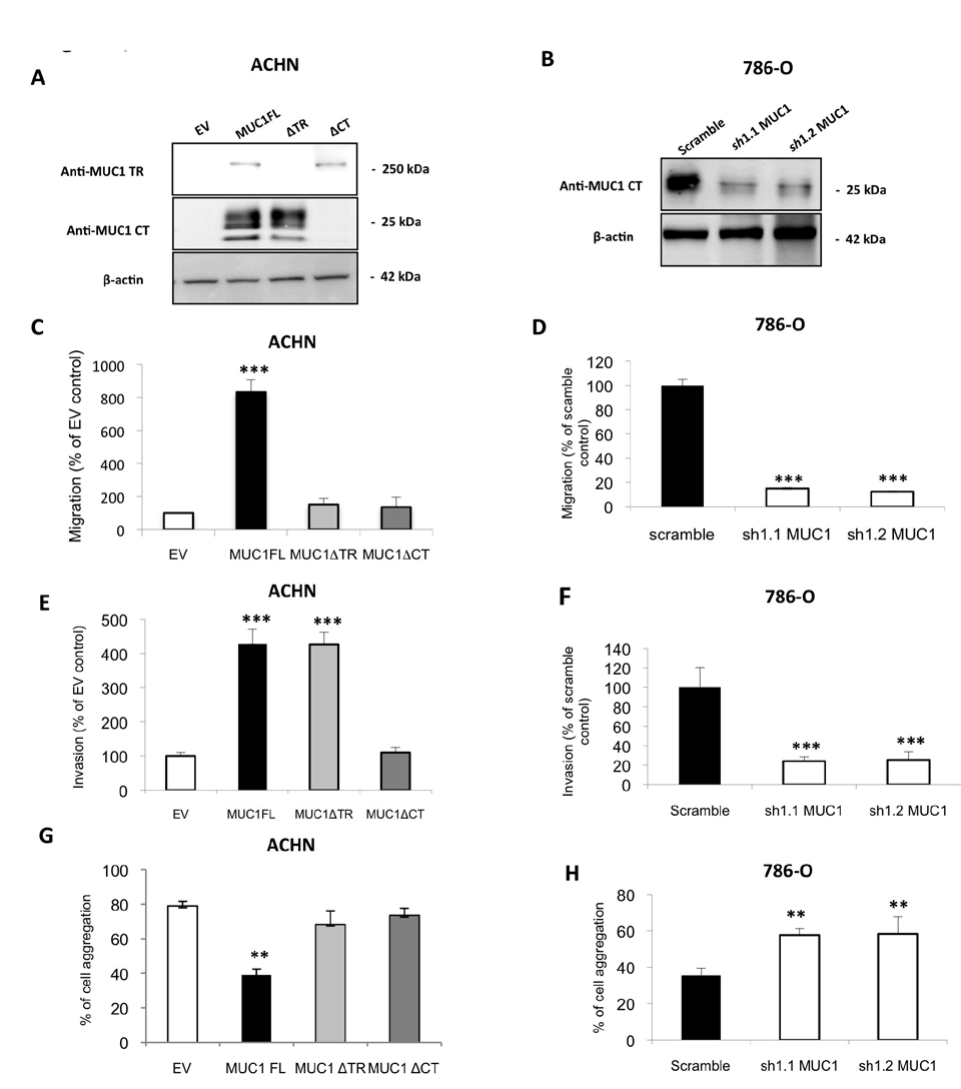
MUC1 increases migratory and invasive properties and decreases cell-cell interaction in ACHN and 786-O cells Western blotting were performed with anti–MUC1 targeting VNTR extracellular domain (M8) or cytoplasmic tail (Ab-5), and anti–β-actin antibodies on whole cell extracts obtained from (A) ACHN clones stably transfected with different expression vectors: MUC1-Full Length (MUC1FL), -deleted for its Tandem Repeat domain (MUC1ΔTR) or -deleted for its Cytoplasmic Tail (MUC1ΔCT) or an empty vector (EV) or (B) from 786-O clones stably transfected with a shRNA control (scramble) or with *sh*RNA targeting MUC1 (*sh*1.1 and *sh*1.2). Cell migration of ACHN (C) and 786-O (D) clones was evaluated using 24-well migration chambers with 10% fetal calf serum as chemoattractant. The values obtained in EV-ACHN and scramble 786-O control cells were referred to as 100. The graphs show a percentage of control migration 24h after seeding. Values are means s.e.m (standard error mean) and represent five separate experiments (*** p<0.001). Cell invasion ((E) ACHN clones and (F) 786-O clones) was evaluated using 24-well Matrigel® invasion chambers with 10% fetal calf serum as chemoattractant. The values obtained in EV-ACHN and scramble 786-O control cells were referred to as 100. The graphs show a percentage of control invasion 24h after seeding. Values are means s.e.m and represent five separate experiments (*** p<0.001). ACHN (G) and 786-O (H) clones were seeded on agarose 0.8% under shaking. After 1h, aggregated and isolated cells were counted to determinate % of cell aggregation. Values are means s.e.m and represent at least three separate experiments (** p<0.01).

**Figure 2:**
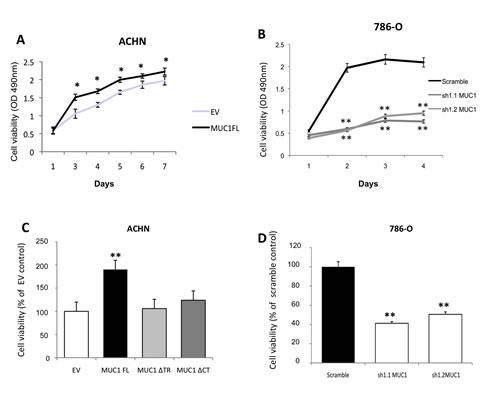
MUC1 increases cellular viability and confers anoikis resistance ACHN (A, C) and 786-O (B, D) clones were seeded in 96-wells plate coated (C, D) or not (A, B) with PolyHema and incubated at 37°C. By a MTS assay, cellular viability was assessed everyday for viability and after 5 days for anoikis. OD was read at 490nm. For anoikis assay, mean in EV-ACHN and 786-O scramble clones was arbitrarily set to 100. Values are means s.e.m and represent five separate experiments (* p<0.05, ** p<0.01).

To assess MUC1 roles on kidney cancer cell properties, we used renal cancer cell lines expressing (786-O) or not (ACHN) MUC1 at protein levels. By stable transfection, we first generated ACHN clones expressing MUC1 full length (MUC1FL; Fig. 1A) and (ii) 786-O clones knock-down for MUC1 expression (MUC1-KD) using a *sh*RNA strategy (*sh*1.1 MUC1 and *sh*1.2 MUC1; Fig. 1B). The role of MUC1 in the migration properties of renal cancer cells was assessed using Boyden chambers (Fig. 1C) and wound healing assays (Fig. 1S); we observed significant enhanced migration in ACHN clones expressing MUC1 by 8.3- and 2-fold, respectively. In contrast, in 786-O MUC1-KD clones, a reduced migration was observed compared to control cells (85% inhibition, p<0.001; Fig. 1D). Invasion assays showed that MUC1 expression significantly enhanced invasiveness properties of ACHN clones compared to EV control cells (4.2 fold, p<0.001; Fig. 1E) whereas MUC1-KD in 786-O lowered invasiveness by 80% (p<0.001; Fig. 1F). MUC1FL ACHN cells presented significantly decreased cell-cell aggregation levels compared to EV ACHN cells (39% *vs* 79%, p<0.01; Fig. 1G) whereas a decreased of MUC1 expression in *sh*1.1 MUC1 and *sh*1.2 MUC1 786-O cells was associated with an increased cell aggregation compared to control cells (59% *vs* 36%, p<0.01; Fig. 1H). The ability of different ACHN and 786-O clones to adhere on type IV collagen, laminin, fibronectin, vitronectin or type I collagen was also assessed but no significant differences were observed for any clone (data not shown). By using a MTS assay, we found that MUC1 expression significantly increased cell viability in MUC1FL ACHN and Scramble 786-O clones (p<0.05 and p<0.01; Fig. 2A and B). Anoikis, an apoptotic program induced by loss of cell-matrix interaction, was finally investigated using poly-HEMA coated plates. After five days, MUC1 expression significantly increased cell viability only in MUC1FL ACHN and Scramble 786-O clones (p<0.01; Fig. 2C and D). Altogether, these results indicate that MUC1 (over)expression in renal cancer cells increases migration, invasion, cell viability, resistance to anoikis and decreases cell-cell interaction. In order to understand the relative contributions of the MUC1 tandem repeat and cytoplasmic tail domains in these properties, we generated by stable transfection ACHN clones expressing MUC1 deleted for its Tandem Repeat domain (MUC1ΔTR) or for its Cytoplasmic Tail (MUC1ΔCT) (Fig. 1A). We showed that both of these domains were essential in migration (Fig. 1C and 1S), cell viability (data not shown), resistance to anoikis (Fig. 2C) and decreased of cell-cell interaction (Fig. 1G) since no significant difference was observed between MUC1ΔTR, MUC1ΔCT and EV-ACHN clones. In sharp contrast, the impact of MUC1 on invasiveness further depends only on MUC1-CT (Fig. 1E) since no difference for invasiveness was observed between EV and MUC1ΔCT ACHN clones.

### MUC1 increases tumor growth *in vivo*

**Figure 3:**
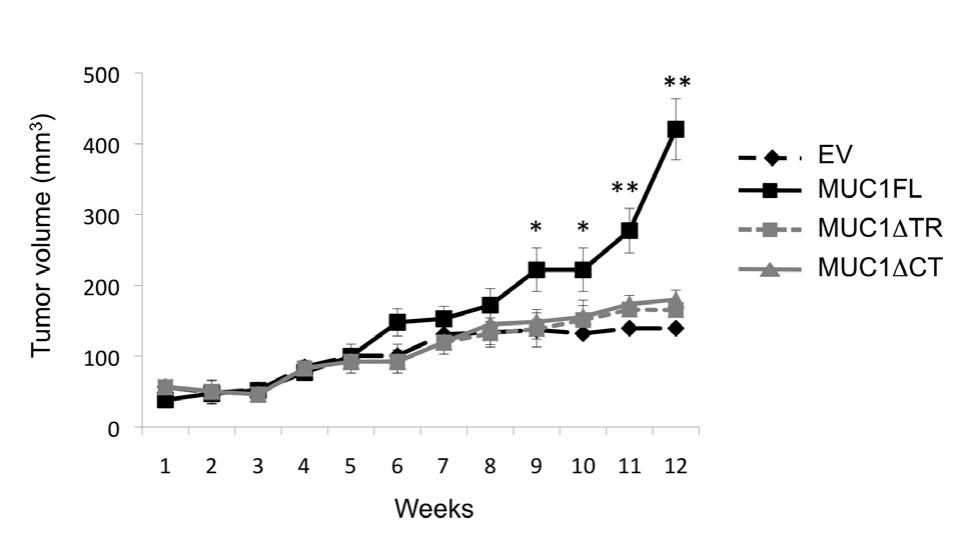
MUC1FL increases cell growth in vivo Subcutaneous injections with ACHN clones stably transfected with an empty vector (EV; 
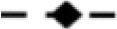
), with MUC1FL- (
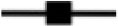
), with MUC1ΔTR- (
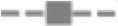
) or with MUC1ΔCT (
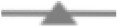
)-expression vectors were performed in SCID mice. Values are means s.e.m and represent values obtained in 6 mice (* p<0.05, ** p<0.01).

To further confirm these *in vitro* data of MUC1 effects on tumor cell properties, subcutaneous xenograft experiments were carried out on SCID mice. From week 9, the tumor volume was significantly higher in xenografted mice with MUC1FL ACHN clones compared to EV control (p<0.05; Fig. 3). At week 12, the relative tumor volume was 420.3 ± 42.9 mm^3^ for MUC1FL clones whereas in control EV-ACHN clones, tumor volume was 139.4 ± 5.7 mm^3^ (p<0.01; Fig. 3). No significant difference was observed between MUC1ΔTR, MUC1ΔCT and EV-ACHN clones. These data show that both tandem repeat domain and cytoplasmic tail of MUC1 are needed for tumor growth *in vivo*.

### Role of MUC1 in intracellular signaling

**Figure 4:**
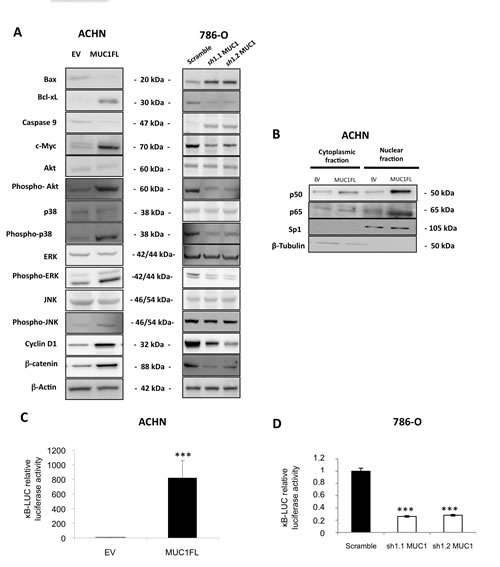
Impact of MUC1 expression on signaling pathways (A) Western blotting on whole cell extracts obtained from EV- and MUC1FL-ACHN clones or from 786-O scramble, *sh*1.1 and *sh*1.2 clones were performed. Antibodies against Bax, Bcl-xL, caspase 9, c-Myc, Akt, phospho-Akt, p38, phospho-p38, ERK, phospho-ERK, JNK, phospho-JNK, cyclin D1, β-catenin and β-actin were used. (B) Expression of NF-κB p65 and p50 subunits in cytosolic and nuclear extracts was carried out by western blot. (C-D) Luciferase activity of the κB-*Luc* synthetic promoter was measured 48h after transfection. Luciferase activity in EV-ACHN and scramble 786-O cells was set as 1. Values are means s.e.m and represent five separate experiments (*** p<0.001).

Having shown that MUC1 expression was associated with an increase of cell viability, migration, invasion, apoptosis resistance and tumor growth, we studied the impact of MUC1 overexpression on the major intracellular signaling pathways by Western blot (Fig. 4A). Expression levels of c-Myc, p-Akt, p-ERK1/2, p-JNK, phospho-p38, cyclin D1 and β-catenin known to be involved in proliferation and tumorigenesis were increased in MUC1 overexpressing MUC1FL ACHN and Scramble 786-O clones compared to EV-ACHN and MUC1-KD 786-O clones.

MUC1 (over)expression in ACHN and 786-O clones was also associated with cell survival since Bax pro-apoptotic marker expression was decreased whereas Bcl-xL anti-apoptotic marker expression was increased resulting in increased of Bcl-xL/Bax ratio suggesting an increase of apoptosis resistance in MUC1 expressing cells. It was also associated with decreased of caspase 9 expression and increased of p50 and p65 NF-κB subunits nuclear localization along with activation of NF-κB anti-apoptotic signaling pathway (Fig. 4B to D). Altogether, these results show that MUC1 expression dramatically increases proliferative, migratory, invasive and anti-apoptotic signaling pathways in renal cancer cells.

### MUC1-C nuclear localization is dependent of ADAM/γ-secretase pathway

**Figure 5:**
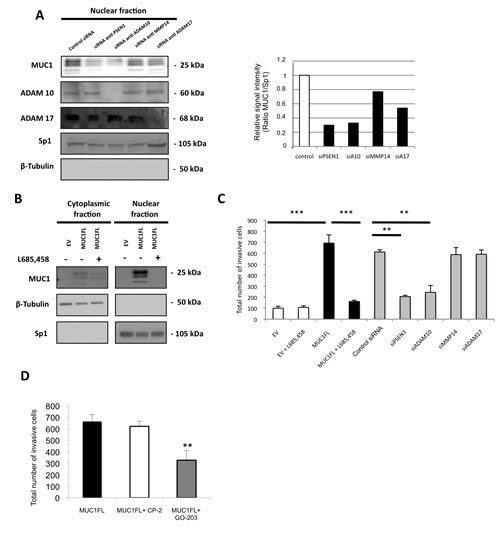
Increase of invasive properties mediated by nuclear MUC1-C is dependent of ADAM10/ADAM17/γ-secretase activities (A) Western blotting was performed on nuclear fraction of ACHN cells after treatment by different *si*RNA. The intensities of the signals were determined by densitometric scanning and are expressed as the relative signal intensity compared with that obtained with control siRNA. (B) Expression of MUC1-CT in cytosolic and nuclear fractions was carried out by western blotting from EV- and MUC1FL-ACHN clones treated or not with 10 ng/ml of L685,458, a γ-secretase inhibitor. (C) Cell invasion of ACHN cells transfected with different *si*RNA or treated 24h with 10 ng/ml of L685,458 was evaluated using 24-well Matrigel® invasion chambers with 10% fetal calf serum as chemoattractant. The graphs show the total number of invasive cells counted 24h after seeding. Values are means s.e.m and represent five separate experiments. (D) Invasion experiment was also performed on ACHN cells treated with 5 μM of CP-2 (control) or GO-203 peptides. Values are means s.e.m and represent five separate experiments (** p<0.01, *** p<0.001)

In normal human epithelial endometrial HES cells, Carson's team has previously shown that (i) MUC1 extracellular domain could be released by a mechanism dependent on the activity of at least two sheddases, TACE/ADAM17 [2] and MT1-MMP/MMP14 [3], and then, (ii) MUC1 transmembrane domain (MUC1-C) was a substrate of the γ-secretase [4]. Herein, we demonstrated a nuclear localization of MUC1-C in renal cancer MUC1 expressing cells (Fig. 5A and 5B) and 786-O scramble clones (data not shown). To test our hypothesis that MUC1-C nuclear localization is dependent of sheddase/γ-secretase pathway, we used a *si*RNA approach (Fig. 5A). Compared to ACHN cells transfected with *si*RNA pool control, MUC1-C nuclear level was strongly reduced in cells in which ADAM10, ADAM17 and PSEN1 (catalytic subunit of γ-secretase complex) expression was knocked-down (65%, 42% and 75% of reduction, respectively; Fig. 5A) whereas *si*RNA pool targeting MMP14 has a weaker effect (22% of reduction). The role of γ-secretase in MUC1-C nuclear localization was confirmed when exposing ACHN cells to L685,458, a γ-secretase inhibitor, since MUC1-C was not detected by Western blotting after 24h treatment (Fig. 5B). In contrast, ACHN cells treatment with epoxomicin (a proteasomal inhibitor) or bafilomycin (a vesicular proton pump inhibitor blocking the endosomal/lysosomal activity) has no impact on MUC1-C nuclear level (data not shown).

Thus, these results indicate that the MUC1-C nuclear localization depends on sheddases (ADAM10 and ADAM17) as well as γ-secretase activities.

### Impact of MUC1-C on invasive properties is dependent on ADAM10/γ-secretase activities

Having shown previously that MUC1-C was enough to increase invasive properties, we tested the hypothesis that the sheddases/γ-secretase pathway could alter the invasiveness of renal cancer cells. By a *si*RNA approach, knockdown expression of PSEN1 and ADAM10 significantly decreased invasiveness compared to MUC1FL ACHN cells transfected with control *si*RNA (70% and 65% of reduction, respectively; Fig. 5C) whereas depletion of MMP14 and ADAM17 has no effect. Also, 24h treatment with L685,458 had no effect on basal invasiveness of EV-ACHN clones whereas in MUC1FL clones, increased invasion mediated by MUC1 expression was almost totally abolished (p<0.001; Fig. 5C). Similar results were observed in 786-O clones (Fig. 2S). However, ADAM10 and γ-secretase have numerous substrates such as Notch and c-Met known to play important roles during renal cancer development [14]. To further define the role of MUC1-C contribution to kidney cancer cell invasiveness, we treated MUC1FL ACHN clones with (i) the MUC1-C inhibitor GO-203, a cell-penetrating D-amino acid peptide which blocks its dimerization and nuclear translocation and (ii) CP-2, a control peptide [15]. Significantly, treatment with GO-203, but not with CP-2, was associated with a 49% decrease of invasiveness (p<0.01, Fig. 5D). Together, these findings indicate that blockage of MUC1-C nuclear localization either through inhibition of ADAM10 and PSEN1 expression or direct MUC1-C inihibitor are enough to significantly decrease renal cancer cell invasiveness.

**Figure 6:**
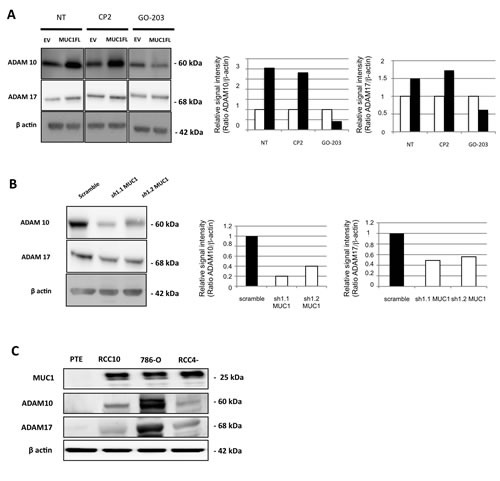
MUC1 is involved in increase of ADAM10 and ADAM17 expression Western blot performed on cell extracts obtained from EV- and MUC1FL-ACHN clones treated or not by 5 μM of CP-2 and GO-203 peptides (A), scramble, *sh*1.1 and *sh*1.2 786-O clones (B) or primary proximal tubular epithelial cells (PTE), RCC4, RCC10 and 786-O cancer cell lines (C). The density of each marker was measured and ADAM10/actin and ADAM17/actin ratios were determined and represented as histograms. Expression in control cells was arbitrarily set to 1.

### MUC1-C induces ADAM10 and ADAM17 expression

**Figure 7:**
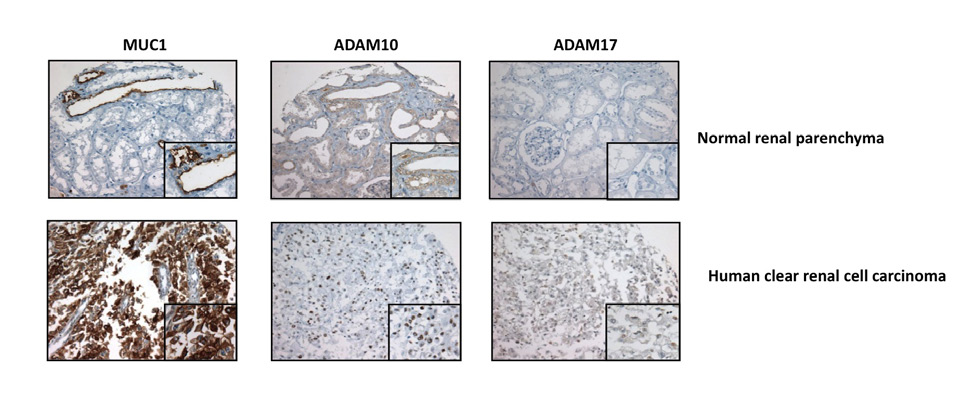
MUC1, ADAM10 and ADAM17 expression in human tissue Immunohistochemical expression of MUC1, ADAM10 and ADAM17 assessed in human normal kidney (up) and a human cRCC (down). In normal kidney, MUC1 staining restricted to apical surface (insert) of distal convoluted tubules, membranous ADAM10 staining is (insert) on distal convoluted tubules and negative ADAM 17 staining. In cCRCC, diffuse cytoplamic MUC1 staining, nuclear ADAM10 staining and focal and weak ADAM17 staining (insert) of tumor cells (Magnification, × 200; x400 (insets)).

Next, we checked expression levels of ADAM10 and ADAM17 in our different cellular clones and showed that in ACHN cells, MUC1 expression was associated with an increased expression of both sheddases by a mechanism depending on nuclear MUC1-C since this increase was lost when MUC1FL clones were treated with GO-203 inhibitor (Fig. 6A). In 786-O cells, MUC1 knocked-down expression mediated a decreased expression of ADAM10 and ADAM17 (Fig. 6B). In normal PTE cells, MUC1, ADAM10 and ADAM17 proteins were undetectable whereas all of them were expressed in RCC4, RCC10 and 786-O renal cancer cell lines (Fig. 6C). By immunohistochemistry, we tested MUC1, ADAM10 and ADAM17 expression on samples of human cRCC. A moderate to strong nuclear expression of ADAM10 was noted in tumor cells presenting a cytoplasmic expression of MUC1 (Fig. 7) while this was faint or absent when MUC1 expression was restricted to the membrane. Comparatively, ADAM17 expression was slightly positive (Fig. 7). In normal renal parenchyma, an apical membranous MUC1 staining was confined to distal convoluted tubules. Immunochemistry for ADAM10 showed a membranous staining pattern in distal tubuli but also in podocytes. No significant staining was observed for ADAM17.

## DISCUSSION

cRCC is the main histotype of kidney cancer, which is typically highly resistant to common systemic therapies. Deciphering molecular mechanisms that lead to tumor progression is urgently needed in order to develop new therapeutic strategies to cure cRCC. In this context, we first focused our attention on MUC1 membrane-bound mucin since its overexpression has been reported in epithelial tumours originating from different tissues, especially in breast and pancreatic tumours [16, 17]. Our team and others have reported that MUC1 (i) is consistently overexpressed in cRCC with a high expression correlated to worse prognosis [11, 18], (ii) cytoplasmic immunostaining is associated with metastatic status [11] and (iii) is a target gene of HIF-1 transcription factor which is a master key of the hypoxia pathway, the main renal carcinogenetic pathway [11].

In this study, to decipher the impact of MUC1 expression on renal cancer cell properties, MUC1 was either ectopically expressed in ACHN cells or knockdown in 786-O cells. We demonstrated that MUC1 significantly increased migration, invasion, cell viability, resistance to anoikis and decreased cell-cell interaction; properties shared with other cancer cell lines such as gastric, pancreatic and breast cancer cells which together suggest a pivotal role of MUC1 to cancer cell phenotype [19-21]. However, little is known about the underlying mechanism. Thus, we showed that both TR and CT domains of MUC1 were necessary for these properties except for invasiveness, which only depended on the CT domain. Overexpression of MUC1 in different cell lines has been shown to inhibit their aggregation by steric hindrance due to its large, extended and rigid structure [22] but also by inhibition of E-cadherin–mediated cell-cell adhesion [23]. MUC1 has also been implicated in cytoskeletal reorganization and cell motility through Src-CrkL-Rac1/Cdc42 signaling cascade following ICAM-1/MUC1 interaction in breast cancer cells [24]. Our signaling pathway studies revealed that MUC1 expression was associated with an increase in the expression of proteins known to be involved in proliferation and tumorigenesis such as c-Myc, phospho-Akt, phospho-ERK1/2, phospho-JNK, phospho-p38, cyclin D1 and β-catenin. Previous reports have shown that down-regulation of MUC1 was followed by an up-regulation of E-cadherin and the relocation of β-catenin from the nucleus to the cytoplasm and a decrease of invasive properties [25, 26]. We also observed that MUC1 overexpression is associated with increased β-catenin protein levels. In fact, MUC1-CT is also considered as a part of the Wnt/β-catenin signaling pathway, which activation is associated to epithelial-mesenchymal transition (EMT), proliferation, migration and invasion in tumor context [1]. MUC1-CT can interact directly with β-catenin through a SXXXXXSSL motif, induces TCF7L2 transcription factor activation and promotes cyclin D1 expression at transcriptional level [27]. Furthermore, MUC1 and β-catenin interaction and their nuclear translocation are able to initiate EMT process, in part by increasing SNAIL transcriptional activity, thus confirming an oncogenic role of MUC1-CT [20, 28]. Roles of MUC1-CT as an intracellular signaling docking molecule as well as a co-transcriptional factor have been well documented since last decade. In sharp contrast, only two recent studies reported that the MUC1 extracellular domain (MUC1-ECD) was found in nuclear speckles and associates with NF-κB p65 and spliceosomes [29, 30]. Further studies are clearly required to better understand new roles of nuclear MUC1-ECD in cancer cells.

Our data also showed that MUC1 expression was associated with cell survival since NF-κB anti-apoptotic signaling pathway was activated and Bcl-xL expression was increased. These results explain, in part, the resistance to anoikis observed in MUC1 expressing cells. Both MUC1-CT and MUC1-ECD were demonstrated to interact with NF-κB p65, then, migrate to the nucleus to activate NF-κB target genes such as Bcl-xL [29, 31]. Additionally, MUC1-CT blocks the interaction between NF-κB p65 and IκBα [31]. Like in Rat 3Y1 fibroblasts, we showed that MUC1 expression activated the anti-apoptotic PI3K/Akt and Bcl-xL pathways and decreased caspase 9 expression [32]. These results combined to numerous published data suggest that MUC1 could be involved in renal cancer chemoresistance since MUC1-overexpressing cancer cells have been pointed out to be unresponsive to chemotoxic agents [33, 34] and to conventional radiotherapy, and that chemotherapy is not effective in cRCC [35].

In the second part of our investigation, we showed for the first time that MUC1-C nuclear localization is instrumental to the invasiness property of renal cancer cells. This mechanism is dependent on both sheddases (ADAM10 and ADAM17) and γ-secretase activities, the blockade of which abolishes the MUC1-C mediated renal cancer cell invasiness. Moreover, as for other substrates of the gamma-secretase pathway, we showed a feedback-loop of sheddase gene expression control by MUC1-C itself. In contrast, pharmacological inhibition of proteasomal or endosomal/lysosomal degradation pathway, known as proteolytic pathways which regulate the degradation of intracellular domain release by the gamma-secretase, had no effect on MUC1-C nuclear level. This result suggests that this MUC1 pool is not in part targeted to degradation but is exclusively dedicated to nuclear signaling. In renal carcinoma context, studies have reported (i) an increased expression of ADAM10 and ADAM17 in renal cancer tissues and cell lines [36, 37] and (ii) that renal cancer cells failed to develop tumours *in vivo* in the absence of ADAM17 [38]. Furthermore, some authors have demonstrated that miR-145 targeted ADAM17 and Oct4 in kidney [37] but also MUC1 in breast cancer cells [39]. MiR-145 expression was decreased in renal carcinoma [37] and downregulated under hypoxic conditions, the main signaling pathway involved in cRCC [40]. Interestingly, cRCC is thought to arise specifically from the epithelial cells of renal proximal tubules [41] and we observed in PTE cells that miR-145 expression is higher than in renal cancer cell lines (Fig. 3S). In contrast, MUC1, ADAM10 and ADAM17 were absent in PTE cells while they were expressed in renal cancer cell lines (Fig. 7C). MiR-145 appears as a differentiation marker and importantly, miR-145, ADAM17 and maybe MUC1 seem to be regulated in a reciprocal negative feedback loop [37]. ADAMs and γ-secretase are a family of proteins that are upregulated in several cancers and represent new therapeutic targets but clinical phase studies of such inhibitors e.g. Batimastat, Marimastat or Semagacestat were discontinued because of side effects such as musculoskeletal toxicity, gastrointestinal symptoms or infections [14, 42]. MUC1-C represents a new attractive druggable target by using promising GO-203 cell-penetrating peptide which blocks MUC1-C dimerization and its nuclear translocation. *In vitro* and *in vivo* studies have shown that GO-203 treatment increased chemosensitivity of cancer cells [43, 44] by decreasing their tumoral properties such as proliferation, resistance to apoptosis [1] and decreased ADAMs expression (this study). This peptide has entered in Phase I evaluation for patients with refractory solid tumors.

In summary, we report, in kidney cancer, that MUC1 is involved in cancer progression and sheddases ADAM10, ADAM17 and gamma-secretase are necessary for MUC1-C nuclear location and in increase of invasiveness. Therefore, we postulate that MUC1/ADAM10/ADAM17 expressions are co-regulated *via* a positive regulatory loop and MUC1 appears as a therapeutic target by blocking MUC1 cleavage or nuclear translocation by using pharmacological approach and peptide strategies.

## MATERIALS AND METHODS

### Cell Culture, RNA interference and transfections experiments

Renal cell lines ACHN and 786-O were obtained from the American Type Culture Collection and cultured in MEM or DMEM supplemented with 10% fetal bovine serum (FBS). The cells were maintained at 37°C under a 5% CO_2_ atmosphere. Primary proximal tubular epithelial (PTE) cells were obtained from human nephrectomy, characterized and maintained as previously described in [45]. Different MUC1 expressing vectors [46] and pRetroSuper.Neo.GFP retroviral vectors encoding small hairpin RNA (shRNA) directed against MUC1 [11] were stably transfected with Effectene® (Qiagen, Courtaboeuf, France) in ACHN and 786-O cells, respectively. Clones were isolated by serial limit dilution. For *si*RNA experiments, ACHN and 786-O cells were seeded the day before transfection at a density of 1 × 10^6^ cells per 75mm^2^ flask in antibiotic-free medium. Cells were transfected with 100 nM of PSEN1, ADAM10, MMP14, ADAM17 or *si*CONTROL^™^ Non-Targeting SMARTpool® siRNA using 60μL of DharmaFECT^™^ transfection reagent according to the manufacturer's instructions (Dharmacon, Perbio, France). In inhibition studies, ACHN and 786-O cells were cultured to 70% confluence and exposed for 24 h to L685,458 (10ng/mL, Calbiochem) or 48 h to 5 μM of GO-203 or CP2 peptides (GenScript). Transient transfection of κB-*Luc* synthetic promoter containing three κB-binding sites was performed with Effectene® (Qiagen) as previously described [47].

### *In vitro* invasion, migration, viability and anoikis assays

Invasion and migration assays carried out as described previously [11]. ACHN and 786-O cells were seeded in 96-well plates coated (anoikis) or not with 50μL of PolyHema (12 mg/mL; Sigma Aldrich, France). For viability and anoikis assays, 20μL of MTS/PMS solution (Promega “Celltiter96® Aqueous Non Radioactive Cell Proliferation Assay” kit) was added daily in each well and placed at 37°C for 3 h. Then, the absorbance was read at 490 nm.

### Aggregation assay

ACHN and 786-O cells were seeded in 6-well plates coated with 0.8% agar and incubated under agitation at 37°C for one hour. Then, isolated and aggregated cells were counted and percentage of cell aggregation was calculated (aggregated cells/ (Isolated cells + aggregated cells) x100).

### Western blotting

Total cellular and nuclear/cytosolic extracts were prepared according to [11]. Western blotting was performed as described previously [11] using specific primary antibodies as detailed in Table S1. For densitometric analysis, the expression level of each protein was carried out using GelAnalyst-GelSmart software (Clara Vision, Paris, France).

### In vivo studies

Subcutaneous injections of 2.10^6^ cells were performed in SCID Beige mice (Charles River, France), bred and maintained under pathogen-free conditions. Tumour growth was followed periodically. The tumour volume (mm^3^) was determined by calculating V = W^2^×L/2 in which W corresponds to the width (in mm) and L to the tumour length (in mm). Mice were killed 55 days after inoculation. All procedures were in accordance with the guidelines and approved by the animal care committee (Comité Ethique Expérimentation Animale Nord Pas-de-Calais, Permit/Protocol number: CEEA 172011).

### Immunohistochemistry

Immunohistochemistry protocols for MUC1 (anti-human: 1/50, MUC1 clone M8; gift from D. Swallow, Imperial Cancer Research, London; anti-rat: 1/500, Muc1 Ab-5, Lab Vision Corp), ADAM 10 (polyclonal anti-human: 1/200, Abcam) and ADAM17 (monoclonal anti-human: 1/200, Abcam) were performed as previously described [11]. IgG control antibodies were used for immunohistochemical analysis and did not show any specific staining.

### Statistical analysis

Statistical analysis of the data was performed using a Student's two-tailed *t*-test. *P*-values <0.05 were considered as significant.

## SUPPLEMENTARY FIGURES AND TABLE




