# Supplementary material for: MUC1-C nuclear localization drives invasiveness of renal cancer cells through a sheddase/gamma secretase dependent pathway
Source: Oncotarget. 2014 Feb 2;5(3):754–63. doi: 10.18632/oncotarget.1768 (PMC3996672; doi:10.18632/oncotarget.1768)
Supplement: Supplementary file 1 [file oncotarget-05-754-s001.pdf]

MUC1-C nuclear localization drives invasiveness of renal cancer cells through a sheddase/gamma secretase dependent pathway - Bouillez et al

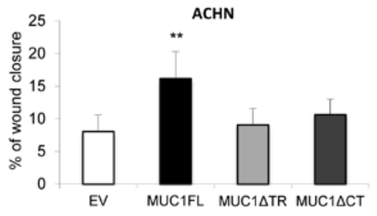

Figure 1S: Extra- and intra-cellular domains of MUC1 increase migratory properties. Confluent monolayers of ACHN cells stably transfected with empty vector or with different constructs of MUC1 were scraped from one side of the reference line. Wounds healing were measured at 0 and 6 hours and percentage of wound closure was determined. Values are means s.e.m and represent five separate experiments (** p<0.01).

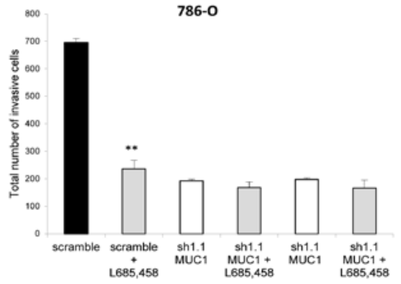

Figure 2S: MUC1-induced invasion in 786-O cells is dependent of γ-secretase activity. Invasion of 786-O cells stably transfected with scramble, *sh*1.1 or *sh*1.2 targeting MUC1 cells was evaluated using 24-well Matrigel® invasion chambers with 10% fetal calf serum as chemoattractant. Cells were treated or not with 10 ng/ml of γ-secretase inhibitor, L685,458. The graphs show the total number of invasive cells counted 24h after seeding. Values are means s.e.m and represent five separate experiments (** p<0.01).

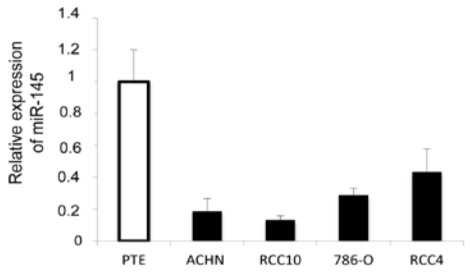

Figure 3S: miR-145 expression is down-regulated in renal carcinoma cells. miR-145 expression was measured in primary proximal tubular epithelial cells (PTE), ACHN, RCC4, RCC10 and 786-O cancer cell lines by Taqman real-time PCR as described in [48, 49]. RNU48 was used as normalizer.
